# Impact of the Kenya post-election crisis on clinic attendance and medication adherence for HIV-infected children in western Kenya

**DOI:** 10.1186/1752-1505-3-5

**Published:** 2009-04-04

**Authors:** Rachel C Vreeman, Winstone M Nyandiko, Edwin Sang, Beverly S Musick, Paula Braitstein, Sarah E Wiehe

**Affiliations:** 1 Children's Health Services Research, Department of Pediatrics, Indiana University School of Medicine, Indianapolis, IN, USA; 2 The Regenstrief Institute, Inc, Indianapolis, IN, USA; 3 USAID – Academic Model Providing Access to Healthcare (AMPATH) Partnership, Eldoret, Kenya; 4 Department of Child Health and Paediatrics, Moi University School of Medicine, Eldoret, Kenya; 5 Department of Medicine, Indiana University School of Medicine, Indianapolis, IN, USA

## Abstract

**Background:**

Kenya experienced a political and humanitarian crisis following presidential elections on 27 December 2007. Over 1,200 people were killed and 300,000 displaced, with disproportionate violence in western Kenya. We sought to describe the immediate impact of this conflict on return to clinic and medication adherence for HIV-infected children cared for within the USAID-Academic Model Providing Access to Healthcare (AMPATH) in western Kenya.

**Methods:**

We conducted a mixed methods analysis that included a retrospective cohort analysis, as well as key informant interviews with pediatric healthcare providers. Eligible patients were HIV-infected children, less than 14 years of age, seen in the AMPATH HIV clinic system between 26 October 2007 and 25 December 2007. We extracted demographic and clinical data, generating descriptive statistics for pre- and post-conflict antiretroviral therapy (ART) adherence and post-election return to clinic for this cohort. ART adherence was derived from caregiver-report of taking all ART doses in past 7 days. We used multivariable logistic regression to assess factors associated with not returning to clinic. Interview dialogue from was analyzed using constant comparison, progressive coding and triangulation.

**Results:**

Between 26 October 2007 and 25 December 2007, 2,585 HIV-infected children (including 1,642 on ART) were seen. During 26 December 2007 to 15 April 2008, 93% (N = 2,398) returned to care. At their first visit after the election, 95% of children on ART (N = 1,408) reported perfect ART adherence, a significant drop from 98% pre-election (p < 0.001). Children on ART were significantly more likely to return to clinic than those not on ART. Members of tribes targeted by violence and members of minority tribes were less likely to return. In qualitative analysis of 9 key informant interviews, prominent barriers to return to clinic and adherence included concerns for personal safety, shortages of resources, hanging priorities, and hopelessness.

**Conclusion:**

During a period of humanitarian crisis, the vulnerable, HIV-infected pediatric population had disruptions in clinical care and in medication adherence, putting children at risk for viral resistance and increased morbidity. However, unique program strengths may have minimized these disruptions.

## Introduction

Conflicts, population displacement, and the economic consequences of disasters affect children disproportionately.[1] Children are more vulnerable to communicable diseases and environmental exposures than adults.[2,3] They have special dietary needs for growth and development, and they are generally dependent on their families.[4] Studies have shown that children under five have the highest mortality rates in conflict-affected settings.[5,6] Furthermore, while acute illnesses and injuries are important in humanitarian emergencies, exacerbation of underlying chronic illnesses can lead to significant morbidity and mortality.[7] When these emergencies occur in the setting of pre-existing poverty, low nutritional status, and immune-compromising diseases such as HIV, children face even greater risks.[8,9]

Little is known about the provision of care for HIV-infected children during complex emergencies. In a small study from an area with long-standing conflict in Uganda, children on ART had high adherence and low mortality.[10] However, there are few guidelines to direct HIV care in these settings,[11] and the optimal methods to coordinate services for conflict-affected populations have seldom been studied. [12-14] For vulnerable pediatric HIV-infected populations, we could not identify any such existing studies. It is essential, therefore, to study the provision of pediatric HIV care in the setting of crisis to determine how HIV-related morbidities and mortality can be prevented or minimized.

Kenya, which has long been one of the most stable and economically developed nations in East Africa, experienced political and humanitarian crises following contested presidential elections held on 27 December 2007. The election results sparked widespread, ethnically related violence and internal displacement of hundreds of thousands of families. By official estimates, over 1,200 people were killed, and over 300,000 people were displaced from their homes.[15] The extent to which the children of Kenya were affected is unknown.

HIV-infected children in Kenya may have been particularly vulnerable during this conflict period. Kenya has over 1.4 million persons (7.8% prevalence) living with HIV (including 150,000 children).[16] As of 30 November 2007, the USAID-Academic Model Providing Access to Healthcare (AMPATH) clinical care system was caring for over 10,000 HIV-infected and exposed children in 17 clinics in western Kenya. Because the western portion of Kenya was severely affected by the violence and displacement of persons,[17] these pediatric patients may have been affected. Thus, we sought to assess the extent to which the Kenya post-election crisis disrupted clinical care and antiretroviral therapy (ART) adherence for HIV-infected children in western Kenya enrolled in AMPATH.

## Methods

### Study Design

We used both quantitative and qualitative techniques to investigate medication and clinic adherence among HIV-infected children in western Kenya before and after the post-election crisis. Using a retrospective cohort design, we assessed changes in adherence using prospectively collected, de-identified clinical data from the computerized medical records of HIV-infected, pediatric patients treated in the AMPATH clinical care system. We complemented these analyses with qualitative key informant interviews of selected healthcare providers who were working within the AMPATH clinical care system during the time of the post-election crisis. We used purposive sampling to identify key informants, including physicians, nurses, and clinical officers, based on their locations and roles during the conflict. A trained facilitator conducted 9 interviews using a prepared, semi-structured interview guide containing open-ended questions. The facilitator solicited information on factors contributing to whether families were able to return to clinic after the elections and on barriers to medication adherence. Furthermore, the quantitative results were presented to the key informants, and they were asked to assess how these results fit with their personal experiences caring for patients during this time period. Thus, qualitative analyses were used both to provide a more in-depth picture of the impact of the post-election crisis on the clinical care system and to corroborate the findings of the database analysis. The participants granted permission to audio-record the interviews. Field notes were also taken during and immediately after the encounters.

### Ethics Statement

The study was approved by the Institutional Research and Ethics Committee of the Moi University School of Medicine and Moi Teaching and Referral Hospital (Eldoret, Kenya) and the Institutional Review Board of the Indiana University School of Medicine (Indianapolis, Indiana). Informed consent was obtained for key informant interviews, and all clinical investigation was conducted according to the principles expressed in the Declaration of Helsinki.

### Study Site

Since 1990, Indiana University School of Medicine has had a collaborative partnership with Moi University School of Medicine in Eldoret, Kenya.[18] AMPATH was created in 2001 as a joint initiative among these two medical schools and Moi Teaching and Referral Hospital to provide an HIV care system for patients in western Kenya. [19-22] AMPATH serves a catchment area of over 13 million people. Since 2001, over 85,000 pediatric and adult patients have been treated within AMPATH, with 14,847 children under the age of 14 years now receiving care and 3,378 children currently on ART (as of 25 February 2009). Comprehensive HIV care services, including the provision of free ART for all qualifying patients, are provided at an urban referral clinic and at 17 rural and outlying outpatient clinics.[20,23] A computerized medical record system supports clinical care and research,[24] and the outcomes and adherence of adult and pediatric patients have previously been reported. [25-27] Clinicians use standard encounter forms at all AMPATH clinic visits , recording information from patient interviews and exams on paper forms. Data from the paper forms are subsequently entered into the AMPATH Medical Record System by dedicated data entry clerks, with data entry validated by random review of 10% of the data entered. This system was designed for use in sub-Saharan Africa, and has proved adaptable in other resource-limited settings, even in the face of challenges such as power outages and supply shortages.[24] The computerized medical record system remained functional throughout the duration of the crisis though the entry of data from paper encounter forms was delayed by several weeks.

### Study Population

Eligible patients included those seen in any of 18 AMPATH clinics between 26 October 2007 and 25 December 2007 (time period 1) who were less than 14 years of age and were HIV-infected. We then followed these children's clinical data from the time of the presidential election (27 December 2007) until 15 April 2008 (time period 2). (The clinics were closed on 26 December 2007.) The pediatric clinics only care for patients less than 14 years of age, so the analyses were restricted to this population. Key informants included physicians, nurses, and clinical officers who were identified by the AMPATH post-crisis evaluation team as having provided clinical care or overseen clinical care for children in AMPATH during time periods 1 and 2. The evaluation team drafted a list of 10 potential interviewees, and all the individuals were approached about their willingness to be interviewed. Nine consented, and one was unavailable.

### Data Collection and Measures

#### Return to Clinic

Return to an AMPATH clinic during time period 2 was captured using appointment data from our electronic medical record system. Children on ART are typically seen on a monthly basis in AMPATH, and HIV-infected children not on ART are seen every two to three months. Thus, all HIV-infected children in our cohort during time period 1 should have had at least one appointment in time period 2. "No Return" to clinic was defined as not having a clinic visit in the time period from 26 December 2007 to 15 April 2008. To assess the extent of loss-to-follow-up that might be expected in a similar cohort over this period of time in a non-conflict period, we also examined clinic appointment data from a comparison group of children from the previous year.

#### ART Adherence

The outcome variable of ART adherence for those children on ART was evaluated from data collected from responses to the question, "During the last 7 days, how many doses of his/her antiretroviral medicines did the patient take?" The response options are: "none," "few," "half," "most," and "all." In this analysis, ART adherence was defined as a binary variable of "imperfect" vs. "perfect" adherence. Patients with imperfect ART adherence (subsequently described as "ART nonadherence") had a visit where adherence was not reported as "all" doses taken during the past seven days (or one or more reports of non-adherence). ART adherence was treated as a binary variable because such high rates of adherence are typically reported in this population and because, among the heterogenous definitions used for adherence in resource-limited settings, this definition is the most common.[28] No validated measure to assess pediatric ART adherence in resource-limited settings currently exists,[28] and this measure has been used in previous studies.[29] Viral loads are not routinely obtained in this clinical care system.

#### Covariates

Other independent variables were selected from the domains of demographic, household, and clinical care information, including child's age, sex, tribe, and in which clinic the child received care. In addition to tribe itself, we also included an indicator variable for patients belonging to a minority tribe that constituted less than 10% of the clinic's population, and orphan status. An orphaned child was defined as one having the mother dead or having both parents dead.

### Analyses

We used descriptive statistics to describe this cohort of children. For the quantitative analysis, we performed multivariable logistic regression analyses to assess factors associated with not returning to clinic (No Return), assessing the independent association between odds of No Return and sex, age, orphan status, clinic site, tribe, being on ART, and belonging to a minority tribe. The standard error was adjusted for correlation within the 18 clinics. We also compared medication adherence rates pre- and post-election using paired t-tests. All models calculated 95 percent confidence intervals based on robust variance estimates. All statistical analyses were performed using Stata/SE 9.2 for Windows (Stata Corp, College Station, TX).

For the qualitative analysis, the audio-recordings and field notes from the key informant interviews were independently reviewed by two investigators. Manual, progressive coding of the field notes and audio-recordings was done to extract themes. Several forms of triangulation were done to increase the credibility of the results. Investigator triangulation was used by involving additional investigators in reviewing the recordings and field notes and in confirming or disconfirming the codes and the subsequent themes. Data triangulation was used by comparing the information reported in the interview dialogue with clinic information recorded by the AMPATH care system about the services provided by individual clinics on each day of the crisis and post-crisis period. Moreover, the use of "mixed methods", in which we combine quantitative and qualitative analyses could also be considered methodological triangulation. The themes extracted from the field notes and recordings were then related to particular portions of the quantitative data that they complemented, contradicted, or explained. Representative quotations were extracted to capture these themes.

## Results

### The context of western Kenya during the post-election crisis period

Western Kenya and Rift Valley, precisely the areas where the AMPATH clinics are located, experienced disproportionate violence and displacement during the weeks following the presidential elections.[17] The AMPATH healthcare providers described the extent of violence and instability. In interviews, pediatric healthcare providers described the trauma children faced during the crisis period:

▪ *There was one boy who was being taken care of by the uncle. They stay in Langas. Langas was, let me say, it was the heat of the violence there. This boy is on second line medication, and at the time of the crisis they tried to travel back to the home, the rural home. He told us he forgot his medication at home. Reaching half of the way, he had forgotten his medication. There was no way he could go back to the house to pick the medication and there was no way he could come to the hospital to pick the medication. And on his way to home, he found dead bodies on the way. Furthermore, he saw a man being hacked by the neck. So when he gave us that terrifying experience, we really got scared. We got touched. And he was telling us that now he missed his second line medication a number of days*.

The healthcare providers were also affected by trauma around them. One described the personal impact and terror of seeing her colleague's home burned down:

▪ *We were all frantic, frightened. Like, I see my neighbor's house burning..."N–'s house is burning!" and you know N– is a nurse in Module X. "N–'s house is burning!" I don't know, we were just screaming...That one has really stuck in me – seeing my colleague's house burn*.

In addition to the witnessed violence, the healthcare providers described being unable to travel from their homes or obtain resources such as food, not being allowed to provide care in particular clinics because of the perceived risk to members of their ethnicity in that community, and experiencing mistrust from patients because of the providers' ethnicity. In the context of this conflict period, the AMPATH clinic system was seen as a place of stability and safety. As one healthcare provider described it, "there was so much trust on the medical side, yet outside was trouble."

The immediate AMPATH response to this humanitarian crisis was multi-faceted. Emergency provision of medicines were given to whomever was able to reach the clinics though staff noted that not having charts or treatment details for all patients sometimes presented a challenge. AMPATH formed an emergency task force that met daily during the immediate crisis period. This team was composed of healthcare providers, administrative staff, and research faculty. On a daily basis, the task force coordinated the staff coverage and resources available for each AMPATH clinic, designated response teams to camps and other locations of internally displaced persons, organized communication with other agencies such as the Kenya Ministry of Health and the International Red Cross, and allocated resources including money, food and HIV testing supplies. Almost all of the clinics were operating within the first week after the elections, but it was not uncommon for clinics to be staffed by only a few healthcare providers, such as a single nurse and clinical officer. AMPATH also established a nationwide hotline to advise patients that included two phone lines that were staffed 24-hours a day to provide instructions on drug use and acquisition, infant feeding, and access to care. AMPATH publicized instructions for HIV-infected patients through radio, newspaper, and local television announcements in both national and local languages. AMPATH also sent teams to the camps for internally displaced persons, satellite clinics and patient homes, where clinical outreach teams provided essential healthcare and medication refills and identified AMPATH patients within camps were enlisted to help trace other patients. Though staff shortages were persistent in some of the clinics throughout this time, the task force organized how to maintain AMPATH's usual comprehensive services by providing food and social support services, in addition to medical care. Most of the HIV clinics were re-opened within the first week of the violence.

### Quantitative Results for Clinical Care Disruption and ART Adherence

**Figure 1:**
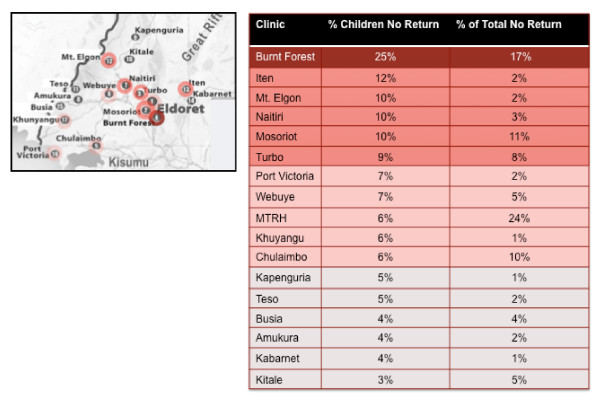
**AMPATH clinic locations and rates of not returning to clinics**.

In the context of this humanitarian crisis and the comprehensive, though impromptu AMPATH response, we examined clinical data for the population of pediatric patients seen in the AMPATH clinics immediately before, during, and after the post-election crisis. In the two months before the presidential elections, between 26 October 2007 and 25 December 2007, 2,585 HIV-infected children were seen in the 17 AMPATH clinics operating during that time period. The median number of children seen in each clinic was 67, with a range of 33 to 769. Of those 2,585 HIV-infected children, 64% (N = 1,642) were on ART. In the immediate months after the presidential election, from 26 December 2007 to 15 April 2008, 93% of these children (N = 2,398) returned to care within the AMPATH clinical care system. Of those who were on ART, 95% returned to care (N = 1,558). The percentages of children not returning to each of the AMPATH clinics are illustrated in Figure 1.

In Table 1, we present the individual characteristics of the children based on return to clinic. A greater proportion of children who returned to clinic were on ART (65%) compared with those who did not return to clinic (45%). The children who did not return to clinic had a lower mean age. For tribal affiliation, A, B, C, and D represent the 4 largest tribe groups seen within the AMPATH clinical care system. Tribe names were not used because of concerns about political sensitivity; however, the letters reflect major tribe groups in Kenya such as Luo, Kalenjin, and Kikuyu. The most prominent difference in the distributions is that only 86% of the children from Tribe D returned to clinic, compared to 92 to 94% of the children from other tribe groups. Tribe D constitutes 8% of the AMPATH pediatric population, but 16% of those with a disruption in return to clinic.

**Table 1:** Patient characteristics based on return to clinic after post-election crisis

	**No Return** N = 187 (column %)	**Returned to Clinic** N = 2,398 (column %)
**Male**	84 (45%)	1,198 (50%)

**On ART**	84 (45%)	1,558 (65%)

**Orphan**	60 (33%)	880 (37%)

**Age**	Median 4.7 yrs	Median 5.9 yrs
	Mean 5.0 yrs	Mean 6.0 yrs
	Standard Dev 3.5	Standard Dev 3.2

**Tribe**		
**A**	51 (27%)	776 (32%)
**B**	51 (27%)	627 (26%)
**C**	30 (16%)	456 (19%)
**D**	30 (16%)	181 (8%)
**Other**	19 (10%)	311 (13%)
**Missing**	6 (3%)	47 (2%)

**Tribe <10% Clinic Population**	55 (30%)	768 (33%)

**Urban Clinic**	45 (24%)	724 (30%)

Table 2 describes the adjusted and unadjusted odds ratios of not returning to clinic by patient characteristics. Looking at the adjusted odds ratios, children who were on ART were significantly more likely to return to clinic (OR = 1.42, 95%CI: 1.22–1.57). Members of Tribe D were significantly more likely to not return to clinic (OR = 2.79, 95%CI: 1.26–6.22), as were children who were members of any tribe that constituted less than 10% of the population at the clinic they attended (OR = 1.33, 95%CI: 1.07–1.51). Orphan status and sex were not associated with return to clinic. The unadjusted odds ratios are similar.

**Table 2:** Odds ratios for not returning to clinic by patient characteristics

	**No Return** **Unadjusted OR (95% CI)**	**No Return** **Adjusted OR (95% CI)**
**Male**	0.82 (0.61–1.09)	0.87 (0.61–1.23)

**On ART**	**0.44 (0.31–0.62)**	**0.58 (0.43–0.78)**

**Orphan**	0.82 (0.61–1.10)	1.03 (0.67–1.55)

**Age (years)**		
<1	1.0 *reference*	1.00 *reference*
1	**0.78 (0.32–1.90)**	0.92 (0.32–2.72)
2	**0.66 (0.33–1.30)**	0.82 (0.36–1.86)
**3**	**0.18 (0.06–0.51)**	**0.26 (0.08–0.80)**
4	**0.41 (0.18–0.97)**	0.55 (0.22–1.38)
5	**0.40 (0.18–0.88)**	0.53 (0.24–1.17)
**6**	**0.32 (0.13–0.77)**	**0.37 (0.15–0.93)**
7	**0.32 (0.14–0.72)**	0.43 (0.18–1.07)
8	**0.22 (0.07–0.66)**	0.34 (0.12–1.01)
**9**	**0.31 (0.17–0.57)**	**0.36 (0.16–0.83)**
**10**	**0.23 (0.10–0.52)**	**0.36 (0.13–0.97)**
11	**0.36 (0.13–0.96)**	0.50 (0.18–1.41)
12+	0.76 (0.32–1.80)	0.98 (0.37–2.64)

**Tribe**		
A	1.0 *reference*	1.00 *reference*
B	1.23 (0.77–1.99)	1.19 (0.72–1.98)
C	1.00 (0.70–1.43)	0.97 (0.62–1.51)
**D**	**2.52 (1.20–5.28)**	**2.79 (1.26–6.22)**
Other	0.93 (0.59–1.47)	0.58 (0.50–1.27)

**Tribe <10% population**	1.28 (0.89–1.53)	**1.33 (1.07–1.51)**

At their last AMPATH visit pre-conflict, 98% of the children on ART (N = 1,490) reported perfect ART adherence during the last 7 days. At their first visit after the election, 95% of the children on ART (N = 1,408) reported perfect ART adherence. Comparing the adherence rates pre- and post-election, significantly fewer children reported perfect ART adherence in the past 7 days when queried during the conflict period (p < 0.001). These figures exclude the 3% of children on ART who were missing data for ART adherence at the pre-conflict visit and the 10% who were missing ART adherence data at the post-election visit; however, the analyses with missing data removed were no different than analyses assuming all those missing data were nonadherent. Thus, the more conservative estimates were used. Data about factors associated with adherence are available upon request.

In comparison, during 26 October 2006 to 25 December 2006, 2,128 HIV-infected children were seen in the AMPATH clinics. Between 26 December 2006 and 15 April 2007, 97% of the children (N = 2,059) returned to care. Of those on ART, 97% children (N = 1,302) returned to care in this non-conflict cohort. Thus, having 93% of the children returning to care during the conflict period was lower than would be expected based on a similar, non-conflict time period in the previous year.

### Perceived Barriers to Returning to Clinic

Healthcare providers within the AMPATH clinical care system uniformly identified fear for personal safety as a major barrier preventing families from returning to clinic in the conflict weeks after the election.

▪ *In the immediate period, January and February, it was safety. Travelling the roads was difficult and unpredictable and very unsafe. And so families felt trapped...Because it was life-threatening for them to go on the roads and try to get [to clinic]*.

The risks to personal safety were seen to vary based on the families' tribal affiliation, their location, and whether they were in the ethnic minority within their location. In particular areas, members of specific tribes were considered targeted for violence, burning of their homes, and forced displacement.

▪ *Typically, the patients who were not to come back were the [Tribe D] people because they were the target. Just fear*.

The lack of resources during this time was another major factor making it difficult for families to return to clinic. The lack of public transportation and roadblocks, which were often manned by armed groups, made travel to clinic difficult. The closure of shops and banks, and subsequent shortages of money, food, and cell phone minutes, added to the challenges. Shortages of resources were seen to disproportionately affect the poorest families, including those caring for orphans.

▪ *The second difficulty, that continued beyond the immediate crisis, was lack of money. No one had transport money and getting to the clinic was so difficult and people had nothing, especially those who had lost homes. They had no resources. And so travelling to the clinic was incredibly difficult*.

Because of the difficulties with finding transportation, healthcare providers described how families who lived or were staying at greater distances from clinic had more difficulty returning to care. Younger children may have been at greater distances from the clinics than older children since they were described as being "like another luggage you carry on your back as you go, as you run and carr [y] to more distant homes."

Despite all of these barriers to returning to clinic, families with children enrolled in AMPATH often assumed huge risks and acted with great bravery to return to the clinic and to obtain their medication refills.

▪ *Patients really surprised us. They walked distances, they ran away, they tried again, they came back. I remember one patient who came from Langas, and he told us it took – it usually takes about 30 minutes to get here, but he took 4 hours to get here. Because he would come, find the road is blocked, run away because the police are shooting or something else is happening, finds houses burning*.

### Perceived Barriers to Medication Adherence

In the key informant interviews, numerous factors were raised as barriers to medication adherence during this crisis period. Safety concerns were again seen as an important barrier. Providers reported that some patients fled from their homes with few possessions, if any, and did not always have their medicines with them. Disruptions in family units sometimes resulted in the absence of a child's usual caregiver or the absence of the family's economic provider. In addition, clinics had shortages of ART medicines or limited availability of the correct pediatric formulations. Furthermore, patients were seen to have changed their priorities. The priorities "became survival and safety" and meeting their basic needs.

▪ *And he was telling me – he was in the Cathedral [IDP camp], he doesn't have a blanket, let alone medication. Because now that was like it is secondary. It was not even a priority. I mean, he is there with nothing*.

Accompanying the perceived change in priorities were particular psychological states that could impact medication adherence. Healthcare providers specifically described how some patients were hopeless, had "general apathy" or had "lost the will to continue living." Other patients were seen as being traumatized or having post-traumatic stress disorder. Hopelessness and being traumatized were thought to decrease adherence to ART.

### Special Considerations Regarding Quantitative Results

While all of the key informant interviewees expressed that the quantitative results fit with their experiences during the conflict period after the elections, they also offered several counterpoints to the findings. First, some interviewees felt that caution was needed because the quantitative results may have overestimated the extent to which clinical care was disrupted. They noted that patients who did not return to clinic may have received clinical care in the camps or shifted to other programs. Interviewees also stressed the heterogeneity between clinic locations, as some clinics were dramatically affected and others were scarcely affected. They further noted that the quantitative data did not adequately capture the bravery of the AMPATH patients or the AMPATH staff. The interviewees emphasized the heightened vulnerability of children during times of crisis, noting children's dependence on the adults around them and how clinic services for children often lagged behind adult services. Finally, the healthcare providers suggested important next steps. They pointed out the ongoing need for outreach efforts to locate, assess, and counsel children missing from clinic.

## Discussion

During a period of widespread violence and displacement of people in western Kenya, some vulnerable, HIV-infected children experienced a breech in clinical care and ART adherence. However, these disruptions were less than had been expected given the intensity of the crisis in the region. While the disruptions in return to clinic and ART provide evidence that HIV-infected children may be at risk for viral resistance, opportunistic infections, and decreased nutrition after humanitarian crises, they also suggest that a comprehensive, responsive HIV care system can mitigate and minimize these disruptions. Children on ART were more likely to return to clinic, possibly reflecting an understanding of the importance of ART adherence. This may highlight the strength of adherence education and support efforts within the AMPATH pediatric clinics. Much of the violence and forcible displacement were reported to occur along lines of tribal affiliation, and, in our clinical data, targeted minority ethnic groups were at highest risk of not returning to clinic.

Although HIV-infected children in western Kenya did face disruptions in clinical care and medication adherence after the presidential elections, the rates of clinical care disruption were lower than what might be expected for a resource-limited setting facing conflict and population displacement. Although outside the scope of this analysis to conclude, it is possible the immediate, multi-faceted AMPATH response to the conflict period decreased the disruptions in clinical care. The AMPATH response was built on an infrastructure of clinics, [18-20] food and medical distribution services, networks of community health workers, and a comprehensive electronic medical record system.[24] The unified attitude and commitment of AMPATH personnel to provide care for all patients were also cited by healthcare providers as key factors enabling an effective response. The combination of existing infrastructure, cohesive and positive staff attitudes, and responsive efforts to find and care for patients may have improved continuity of clinical care and ART.

This study has several limitations that merit consideration. First, while the 3% drop in reported ART adherence was a statistically significant difference, it is difficult to know the clinical significance in a setting where viral loads and resistance testing are not routine. The AMPATH pediatric population generally reports very high levels adherence, particularly when monitored over a short period of time.[30] Thus, even a relatively small drop in ART adherence may have clinical significance when contrasted to the very high rates of adherence routinely reported. Furthermore, this was a very conservative measure of nonadherence that may have missed early episodes of nonadherence prior to the patient's return to clinic. Second, even with relatively high estimates of return to clinic and medication adherence, the data likely underestimate the extent to which patients received clinical care. In the first weeks after the election, many of the patients who made it to a clinic were given medication refills for themselves and even their entire families without any record-keeping. Paper encounter forms may not have been filled out, or data entry may have been incomplete. The increase in missing data in the post-election period may reflect both staff shortages and shifting care priorities in the clinic system during the crisis period. Some patients also had an excess drug supply over the holiday season. Moreover, data from visits done by AMPATH teams in the camps or other impromptu sites, as well as data from unaffiliated HIV programs are not included. However, our analyses do include a long follow-up period that would likely extend beyond the first visits and the extra medication supplies. Furthermore, since few other clinical sites in western Kenya provide free ART, the other options for patients to obtain medications were somewhat limited. AMPATH has ongoing initiatives to find patients lost to follow-up from the clinic system. These data were also limited to the information populated in the pediatric electronic medical record. Thus, we could not assess additional, potentially important variables if they were not collected on the routine clinical encounter forms, such as displacement from homes. Assessing these additional contextual factors affecting children remains an important target for the AMPATH clinical system. The key informant interviews provided information about the crisis impact from the perspective of the healthcare providers, but not necessarily from the perspective of families and children. In-depth exploration of the longer-term psychological and social impact of the election conflict on individual children is still needed and is ongoing within the AMPATH clinical care system. Still, this qualitative analysis does provide insight into the factors impacting medication adherence and return to clinical care from the personnel who were the care system's first responders during the time of crisis and thus reflects the immediate experiences within the care system. Finally, both the quantitative and qualitative data rely on the experiences of subjects in a very particular part of the world and in a unique political situation, limiting the generalizability of the results. AMPATH is considered a model of care in under-resourced settings,[19,31] so return to clinic and ART adherence may be much more impacted in care systems that do not provide similar comprehensive, responsive services. Furthermore, the barriers to return to clinic and adherence are consistent with those identified in research from other conflict settings.[14] Because only limited data are available to describe the impact of crises and conflicts on pediatric HIV care, these data from Kenya provide an important addition to understanding how HIV care systems and humanitarian aid organizations can meet the needs of HIV-infected children in future crises.

## Conclusion

In conclusion, this mixed methods study underscores the risks for HIV-infected children during humanitarian crises, while offering some suggestion that comprehensive, responsive clinical care systems can minimize these risks even during very fraught circumstances. While this analysis is somewhat limited by the methodological constraints of a retrospective cohort analysis of clinical data, it does provide timely data for a vulnerable population that has rarely been studied. These data suggest that HIV-infected children are, indeed, at risk for treatment interruptions during crises. We would highly recommend that HIV care programs and relief agencies develop advance plans to minimize disruption of HIV care services during humanitarian crises, including plans for locating children lost to follow up, finding methods to distribute ART closer to patients' homes, and mobilizing care coordination teams. In addition, patients may need monitoring for subsequent opportunistic infections and viral resistance.

## Abbreviations

AMPATH: USAID – Academic Model Providing Access To Healthcare; ART: Antiretroviral therapy; HIV: Human Immunodeficiency Virus.

## Competing interests

Although this work was funded, in part, by the United States Agency for International Development as part of the President's Emergency Plan for AIDS Relief (PEPFAR), this funding source had no role in study design; in the collection, analysis, or interpretation of data; in the writing of the report; or in submission decisions.

None of the authors have any competing interests to disclose.

## Authors' contributions

RCV had full access to all the data in the study and had final responsibility for the decision to submit for publication. RCV conceived of the study, participated in its design and coordination, conducted the key informant interviews, did the qualitative and quantitative analyses, drafted the manuscript, and approved the final manuscript. WMN contributed to the conception and design of the study and participated in the acquisition of data and qualitative analyses. He revised the manuscript critically and gave final approval for publication. ES and BM organized the study data, contributed to the conception and design of the study, revised the manuscript critically, and gave final approval for publication. PB contributed to the conception and design of the study, revised the manuscript critically, and gave final approval for publication. SEW contributed to the conception and design of the study, conducted and supervised the qualitative and quantitative analyses, provided extensive critical revision to the manuscript, and gave final approval of the version to be published.

## Authors' informations

RCV is an Assistant Professor of Pediatrics at the Indiana University School of Medicine in the Division of Children's Health Services Research and Co-Director of Pediatric Research for the Academic Model Providing Access to Healthcare (AMPATH) in western Kenya. She is also a Faculty Investigator with the Center for Health Policy and Professionalism Research at the Indiana University School of Medicine and an Affiliated Scientist at the Regenstrief Institute, Inc. WMN is a Senior Lecturer in the Department of Child Health and Paediatrics at Moi University School of Medicine and Associate Program Manager for the AMPATH partnership, serving as the Co-Director for the AMPATH research network. He is also the Pediatrician-In-Charge for the AMPATH Pediatric HIV Care Program and the Neonatal Unit of Moi Teaching and Referral Hospital. ES is a Data Manager for AMPATH, based in Eldoret, Kenya. BSM is a Data Manager in the Department of Medicine, Indiana University School of Medicine. PB is an Assistant Professor of Medicine at the Indiana University School of Medicines and Co-Field Director of Research for AMPATH. SEW is an Assistant Professor of Pediatrics at the Indiana University School of Medicine, Faculty Investigator with the AMPATH Pediatric Research Working Group, and an Affiliated Scientist at the Regenstrief Institute, Inc.
